# Dosimetric comparison of VitalBeam^®^ and Halcyon^TM^ 2.0 for hypofractionated VMAT with simultaneous integrated boost treatment of early‐stage left‐sided breast cancer

**DOI:** 10.1002/acm2.13428

**Published:** 2021-09-23

**Authors:** Eunbin Ju, Eun Jeong Heo, Chun Gun Park, Minseok Kim, Kwang Hyeon Kim, Jang Bo Shim, Young Je Park, Nam Kwon Lee, Chul Yong Kim, Suk Lee

**Affiliations:** ^1^ Department of Radiation Oncology College of Medicine Korea University Seoul Korea; ^2^ Department of Bio‐Medical Science Graduate School of Korea University Sejong Korea; ^3^ Department of Mathematics Kyonggi University Gyeonggi Korea; ^4^ Department of Biostatistics and Computing Yonsei University Graduate school Seoul Korea; ^5^ Department of Neurosurgery Ilsan Paik Hospital College of Medicine Inje University Goyang Korea; ^6^ Department of Radiation Oncology Guro Hospital Korea University Medical Center Seoul Korea

**Keywords:** dosimetric comparison, Halcyon^TM^, left‐sided breast cancer, VitalBeam®

## Abstract

**Purpose:**

This study compared the quality of treatment plans for early‐stage, left‐sided breast cancer, as planned for and delivered by the Halcyon^TM^ and VitalBeam^®^.

**Materials and methods:**

Fifteen patients diagnosed with early‐stage left‐sided breast cancer, who had received VMAT with hypofractionated SIB, were recruited. All cases were planned using Halcyon^TM^ comprising a dual‐layer MLC (DL‐MLC) and VitalBeam^®^ with a Millennium 120 MLC (VB‐MLC). For the PTVs, the quality of coverage (QC), conformity index (CI), and homogeneity index (HI) were calculated for each plan. The dosimetric differences between the two treatment plans were statistically compared using the Wilcoxon signed‐rank test (*p* < 0.05). To evaluate delivery efficiency, the average delivery time for each patient's treatment plan was recorded and compared.

**Results:**

For the PTVs, the two plans (DL‐MLC and VB‐MLC) were comparable in terms of the QC, CI, and HI. However, *V*_30Gy_ and *D*_mean_ for the heart in the DL‐MLC plan were significantly reduced by 0.49% and 14.6%, respectively, compared with those in the VB‐MLC plan (*p* < 0.05). The *D*_mean_ value for the ipsilateral lung in the DL‐MLC plan significantly decreased by 5.5%, compared with that in the VB‐MLC plan (*p* < 0.05). In addition, the delivery times for the DL‐MLC and VB‐MLC plans were 79 ± 10 and 101 ± 11 s, respectively.

**Conclusions:**

DL‐MLC plans were found to improve OAR sparing. In particular, when treating left‐sided breast cancer via DL‐MLC plans, the risk of heart toxicity is expected to be reduced.

## 1 INTRODUCTION

Breast cancer is the most commonly diagnosed cancer in women worldwide. The American Cancer Society estimated that there were 2 261 410 new female breast cancer cases in 2020, accounting for 11.7% of the total cancer cases in 2020.^1^ The recent decline in breast cancer mortality rates might be attributed to early detection with mammography, adjuvant systemic therapies, and regional radiotherapy.^2^ Radiotherapy after breast‐conserving surgery not only substantially reduces the risk of local recurrence, but also moderately reduces the risk of death from breast cancer and increases long‐term survival compared with mastectomy or lumpectomy alone.^3^, ^4^

After breast‐conserving therapy, dose escalation with the addition of a boost to the tumor bed could reduce local recurrence^5^; however, it extends the duration of treatment and increases the risk of breast fibrosis.^6^ Nevertheless, the simultaneous integrated boost (SIB) technique, whereby doses are delivered at a high dose per fraction to the tumor bed, leads to minimization of the overall treatment time. This technique also has several dosimetric benefits regarding dose conformity to the target and reduction of dose per fraction to organs at risk (OARs).^7^, ^8^ Furthermore, advanced radiotherapy techniques such as intensity‐modulated radiotherapy (IMRT) and volumetric modulated arc therapy (VMAT) improve the target coverage while exposing OARs to lower dose volumes.^9^

Radiation exposure of the lungs is unavoidable when irradiating breasts with or without regional lymph nodes, and increases the risk of pulmonary radiation toxicity, such as the risk of radiation‐induced pneumonitis and pulmonary fibrosis.^10^, ^11^, ^12^ Furthermore, presumably through incidental irradiation of the heart, the risk of ischemic heart disease can be increased by radiotherapy.^13^, ^14^ Thus, when treating breast cancer, it is crucial to reduce incidental doses to the heart and lungs using modern radiotherapy techniques. These efforts could lead to a reduction in the risk of radiation‐induced toxicity.^15^

The linear accelerator called Halcyon that features the MLC was introduced by Varian Medical Systems (Palo Alto, CA). This system features a dual‐layer stacked and staggered MLC with a 1‐cm leaf width in each layer and is configured to simultaneously provide sufficient beam modulation and attenuation.^16^, ^17^ Although it has larger leaf widths than the Millennium 120 MLC, Halcyon^TM^ has an increased gantry speed, leaf speed, and decreased dosimetric leaf gap (DLG) compared with VitalBeam^®^ (Varian Medical Systems, Palo Alto, CA, USA).

This study compared the quality of treatment plans for early‐stage, left‐sided breast cancer, as planned for and delivered by the Halcyon^TM^ and VitalBeam^®^.

## 2 MATERIALS AND METHODS

### 2.1 Patient characteristics

Fifteen patients, who were diagnosed with early‐stage left‐sided breast cancer and had been treated with VMAT‐hypofractionated SIB technique with VitalBeam after conservative breast surgery from our institution, were included in this study. The study was conducted between January 2018 and January 2019.

### 2.2 Treatment systems

The mechanical characteristics of the Halcyon^TM^ and VitalBeam^®^ treatment systems are shown in Table 1 in detail. VitalBeam^®^ is configured with multiphoton beam energy (6 MV, 10 MV, 6 MV‐FFF). The Millennium 120 MLC system used in VitalBeam^®^ features the MLC speed of 2.5 cm/s and transmission of 1.36%. The system with gantry speed is 1 rpm.

**TABLE 1:** Mechanical characteristics of Halcyon^TM^ and VitalBeam^®^

Parameter	Halcyon^TM^	VitalBeam^®^
**Photon energy**	6 MV FFF only	6, 10 MV, 6 MV FFF
**Maximum field size**	28 cm × 28 cm	40 cm × 40 cm
**MLC design**	Dual‐layer MLC	120 MLC
**Leaf width (effective leaf width)**	10 mm (5 mm)	5 mm (5 mm)
**DLG**	0.01 mm	0.5 mm
**MLC speed**	5.0 cm/s	2.5 cm/s
**Gantry speed**	4 rpm	1 rpm
**Nominal 6FFF transmission**	Single‐layer: 0.47% Dual‐layer: 0.01%	1.36%

DLG, dosimetric leaf gap; FFF, flattening‐filter‐free.; MLC, multileaf collimator.

Halcyon^TM^ is a ring‐type radiation treatment delivery system that offers only a 6‐MV flattening‐filter‐free (FFF) photon beam with the maximum dose rate of 800 MU/min. The omission of a flattening filter provides a higher dose rate and lower average energy than that of a 6‐MV photon beam flattened with a filter.^16^ Another feature of Halcyon^TM^ is the new dual‐layer stacked MLC system with the speed of 5.0 cm/s, which is faster than the speed of the Millennium 120 MLC. This DL‐MLC design with faster MLC speed significantly reduces the leakage and transmission: 0.01% transmission for the DL‐MLC compared with 1.36% for the Millennium 120 MLC.^18^

### 2.3 Planning condition

CT scans were acquired under free breathing using a Big Bore CT scanner (Philips Healthcare, Cleveland, OH, USA). The clinical target volume (CTV) delineation was performed by physicians based on the ESTRO consensus guideline.^19^ The planning target volume (PTV) was defined with the CTV expansion of 5 mm and cropped by 5 mm inside the patient outline to exclude the derma region. The boost volume was defined according to the surgical bed, determined by adding 1 cm to the surgical clips placed in the lumpectomy cavity during surgery.

VB‐MLC plans were originally established and DL‐MLC plans were retrospectively established in the Eclipse TPS (version 15.6; Varian Medical Systems, Palo Alto, CA, USA) using the same planning condition (Table 2). The same isocenter was used across the two systems. The prescription dose was 4256 cGy for the PTV, with a 266 cGy daily dose and 5248 cGy for the integrated boost volume, with the daily dose of 328 cGy. All plans were optimized using the Eclipse photon optimization algorithm, and calculated using an analytic anisotropic algorithm, to deliver at least 95%–105% of the prescribed dose to the targets.

**TABLE 2:** Treatment planning conditions applied to both Halcyon^TM^ and VitalBeam^®^

Parameter	Photon energy	Prescription dose (PTV)	Prescription dose (Boost)	Fractions	Coll. Angle
**Value**	6 FFF	4256 cGy	5248 cGy	16 fr	140–300 (2 arc)

PTV, planning target volume.

The considered OARs were the heart, ipsilateral and contralateral lungs, and the contralateral breast. The dose constraints were established based on our institutional guidelines (heart *V*_30Gy _< 5%, *D*_mean _< 500 cGy; contralateral breast *D*_mean _< 300 cGy; ipsilateral lung *D*_mean _< 1000 cGy; contralateral lung *D*_mean _< 200 cGy; whole lung *V*_20Gy_ < 15%, *D*_mean _< 800 cGy).

### 2.4 Evaluation of plan quality and delivery time

In terms of the PTV, the quality of coverage (QC), conformity index (CI), and homogeneity index (HI) were evaluated for the treatment plans, as described in Table 3.^22^, ^23^ For OAR sparing, heart *V*_30Gy_, heart mean dose (*D*_mean_), whole lung *V*_20Gy_, whole lung mean dose (*D*_mean_), right (Rt.) breast mean dose (*D*_mean_), and Rt. lung mean dose (*D*_mean_) were evaluated.

**TABLE 3:** Plan quality evaluation indices with corresponding formula in terms of PTV

Plan evaluation index for PTV	Formula^22^, ^23^
Quality of coverage	*I*_min_/RI
Conformity index	PIV/TV
Homogeneity index	*I*_max_/RI

*I*_max_, maximum isodose surrounding the target; *I*_min_, minimal isodose surrounding the target; PIV, prescription isodose volume; RI, reference isodose, 95% isodose; TV, target volume.

To evaluate the delivery time, the actual beam‐on time was compared between the DL‐MLC and VB‐MLC plans for 15 patients.

### 2.5 Statistical evaluation

The Wilcoxon signed‐rank test, which is a nonparametric statistical hypothesis test, was used to compare the two treatment plans, corresponding to the Halcyon^TM^ and VitalBeam^®^ systems, for matched patients.

## 3 RESULTS

### 3.1 Analysis with isodose distribution and DVH

**FIGURE 1:**
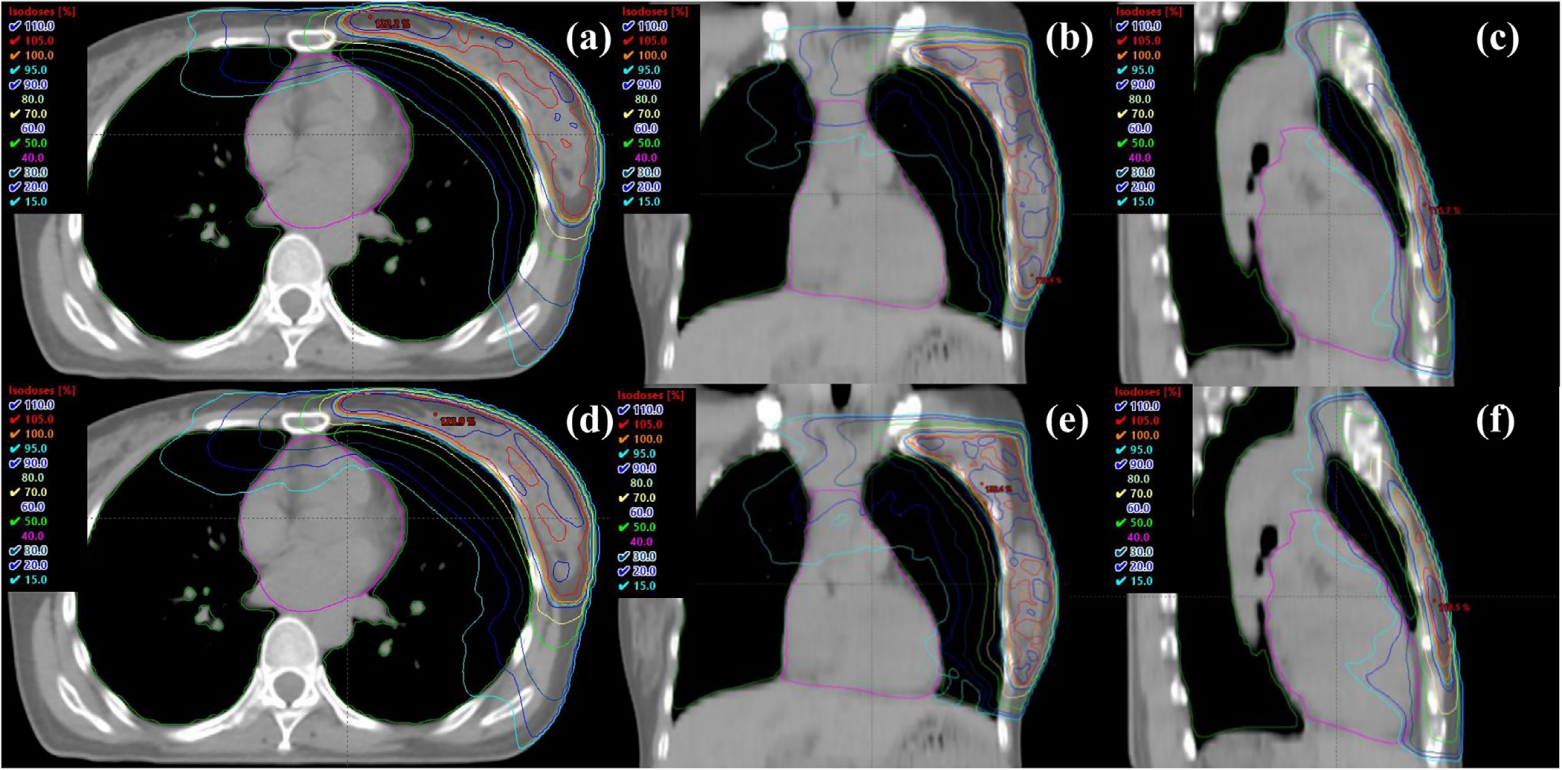
Dose distributions for the example case of comparison between dual‐layer MLC (DL‐MLC) and Millennium 120 MLC (VB‐MLC) plans for early‐stage left‐sided breast cancer. Representative isodose distributions of the DL‐MLC plan in (a) axial, (b) coronal, and (c) sagittal plane. Representative isodose distributions of the VB‐MLC plan in (d) axial, (e) coronal, and (f) sagittal plane. The blue, red, orange, cyan, dark green, yellow, green, purple, blue, and light blue isodose lines represent 110%, 105%, 100%, 95%, 90%, 70%, 50%, 30%, 20%, and 15% isodose, respectively

The dose distributions in the axial, coronal, and sagittal planes for the DL‐MLC and VB‐MLC plans are shown in Figure 1 (Patient 6). More doses were generated for the VB‐MLC plan than for the DL‐MLC plan. The isodose lines correspond to 110% (blue), 105% (red), 100% (orange), 95% (cyan), 90% (dark green), 70% (yellow), 50% (green), 30% (purple), 20% (blue), and 15% isodose (light blue).

**FIGURE 2:**
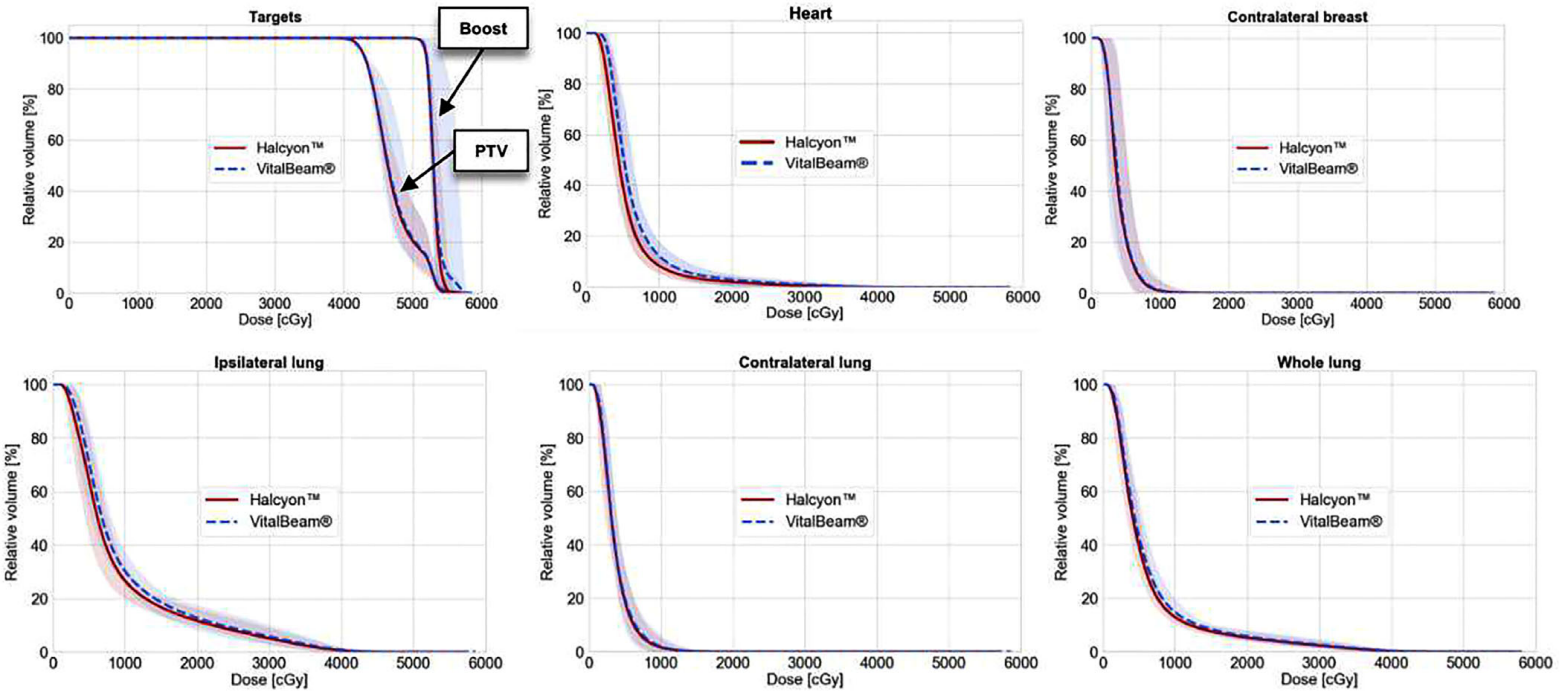
Average dose–volume histogram (DVH) for the target volumes and the main organs at risk (OARs) for the left‐sided breast cancer patients. The solid line and shaded bands represent the average and min–max range of the DVHs across all 15 patients in the planning study

Figure 2 shows the average dose–volume histogram (DVH) of the 15 left‐sided breast cancer patients for the target volumes and OARs. The solid line and shaded bands represent the average and min–max range of the DVHs across all 15 patients in the planning study.

### 3.2 Analysis of plan quality and delivery time between DL‐MLC and VB‐MLC plans

**FIGURE 3:**
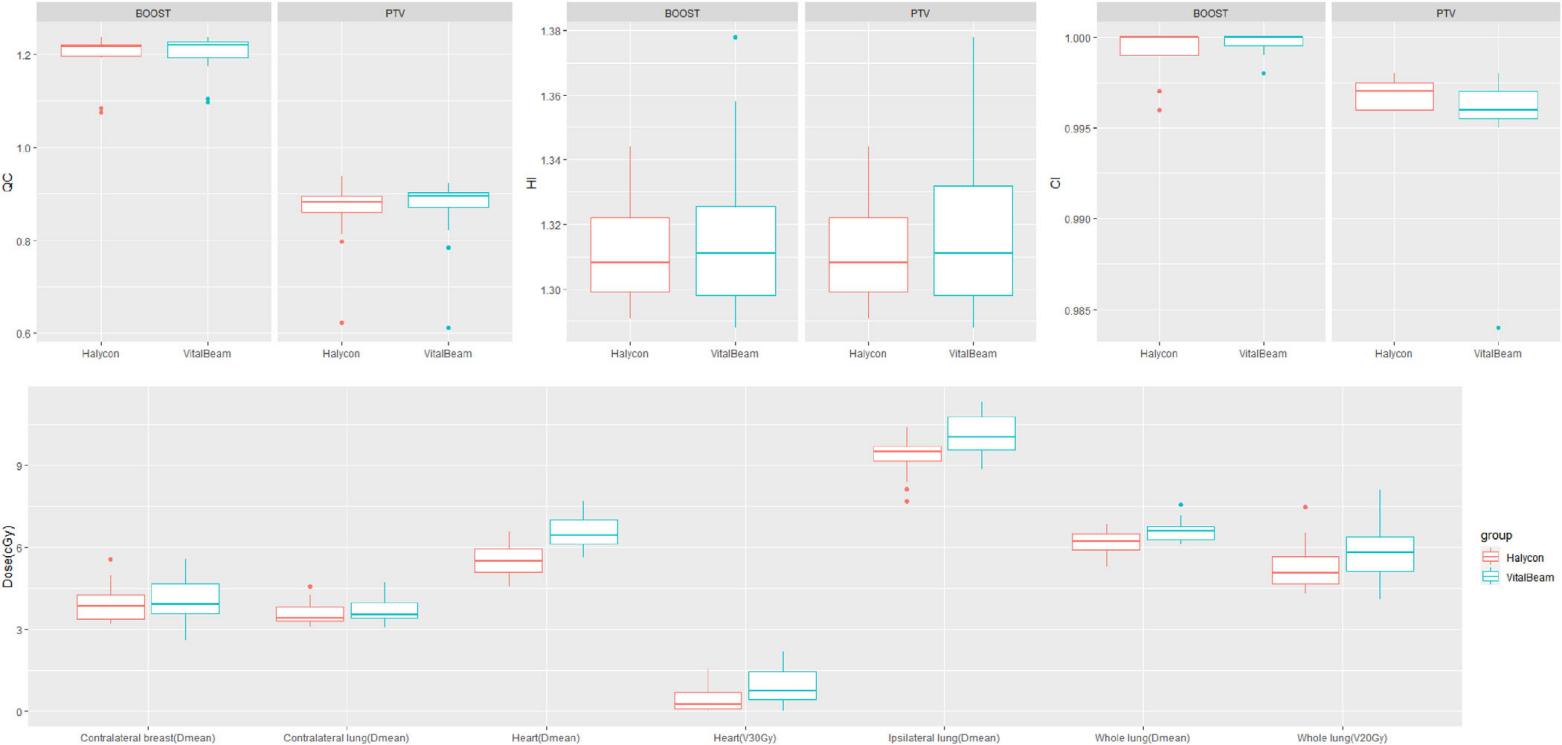
Results of analysis of plan quality evaluation index for comparison of DL‐MLC with VB‐MLC plans

Figure 3 and Table 4 show the results of the plan quality evaluation index for comparison between the DL‐MLC and VB‐MLC plans. Considering targets, DL‐MLC plans had similar target coverage compared with VB‐MLC plans, and there was no significant difference in the QC (0.881 ± 0.076, 0.849 ± 0.075), HI (1.308 ± 0.016, 1.311 ± 0.027), and CI (0.997 ± 0.001, 0.996 ± 0.003) between the DL‐MLC plans and VB‐MLC plans (*p* > 0.05).

**TABLE 4:** Results of evaluation index for comparison between DL‐MLC and VB‐MLC plans

Structure	Plan evaluation index	Halcyon^TM^ (median ± SD)	VitalBeam^®^ (median ± SD)	*p* Value
PTV	QC	0.881 ± 0.076	0.849 ± 0.075	0.639
	CI	0.997 ± 0.001	0.996 ± 0.003	0.034
	HI	1.308 ± 0.016	1.311 ± 0.027	0.615
Boost	QC	1.215 ± 0.049	1.220 ± 0.045	0.073
	CI	1.000 ± 0.001	1.000 ± 0.001	0.102
	HI	1.308 ± 0.016	1.311 ± 0.027	0.762
Heart	HI	0.256 ± 0.498	0.746 ± 0.697	*<0.001* **
	*V*_30Gy_ (%)	549.024 ± 61.894	642.656 ± 65.445	*<0.001* **
Contralateral breast	*D*_mean_ (cGy)	383.040 ± 72.563	391.552 ± 83.361	0.326
Ipsilateral lung	*D*_mean_ (cGy)	949.088 ± 74.144	1004.416 ± 74.652	*<0.001* **
Contralateral lung	*D*_mean_ (cGy)	340.480 ± 44.747	353.248 ± 44.836	0.176
Whole lung	*D*_mean_ (cGy)	5.065 ± 0.888	5.823 ± 1.060	*0.010* *
	*V*_20Gy_ (%)	621.376 ± 42.687	659.680 ± 39.908	*<0.001* **

CI, conformity index; HI, homogeneity index; QC, quality of coverage.

* *p* < 0.05.

** *p* < 0.001.

*V*_30Gy_ and *D*_mean_ for the heart were significantly lower in the DL‐MLC plans than in the VB‐MLC plans (*V*_30Gy_ = 0.256% ± 0.498%, 0.746% ± 0.697%, *p* < 0.001, *D*_mean_ = 549.024 ± 61.894, 642.656 ± 65.445 cGy, *p* < 0.001). The *D*_mean_ value for the ipsilateral lung was significantly lower in the DL‐MLC plans than in the VB‐MLC plans (*D*_mean_ = 949.088 ± 74.144, 1004.416 ± 74.652 cGy, *p* < 0.001). *V*_20Gy_ and *D*_mean_ of the whole lung were significantly lower in the DL‐MLC plans than in the VB‐MLC plans (*V*_20Gy_ = 5.065% ± 0.888%, 5.823 ± 1.060%, *p* = 0.010, *D*_mean_ = 621.376 ± 42.687, 659.680 ± 39.908 cGy, *p* < 0.001). The *D*_mean_ values of the contralateral breast and contralateral lung for DL‐MLC plans were lower than those for the VB‐MLC plans (contralateral breast: 383.040 ± 72.563, 391.552 ± 83.361 cGy; contralateral lung: 340.480 ± 44.747, 353.248 ± 44.836 cGy). However, these differences are not statistically significant (*p* > 0.05).

**FIGURE 4:**
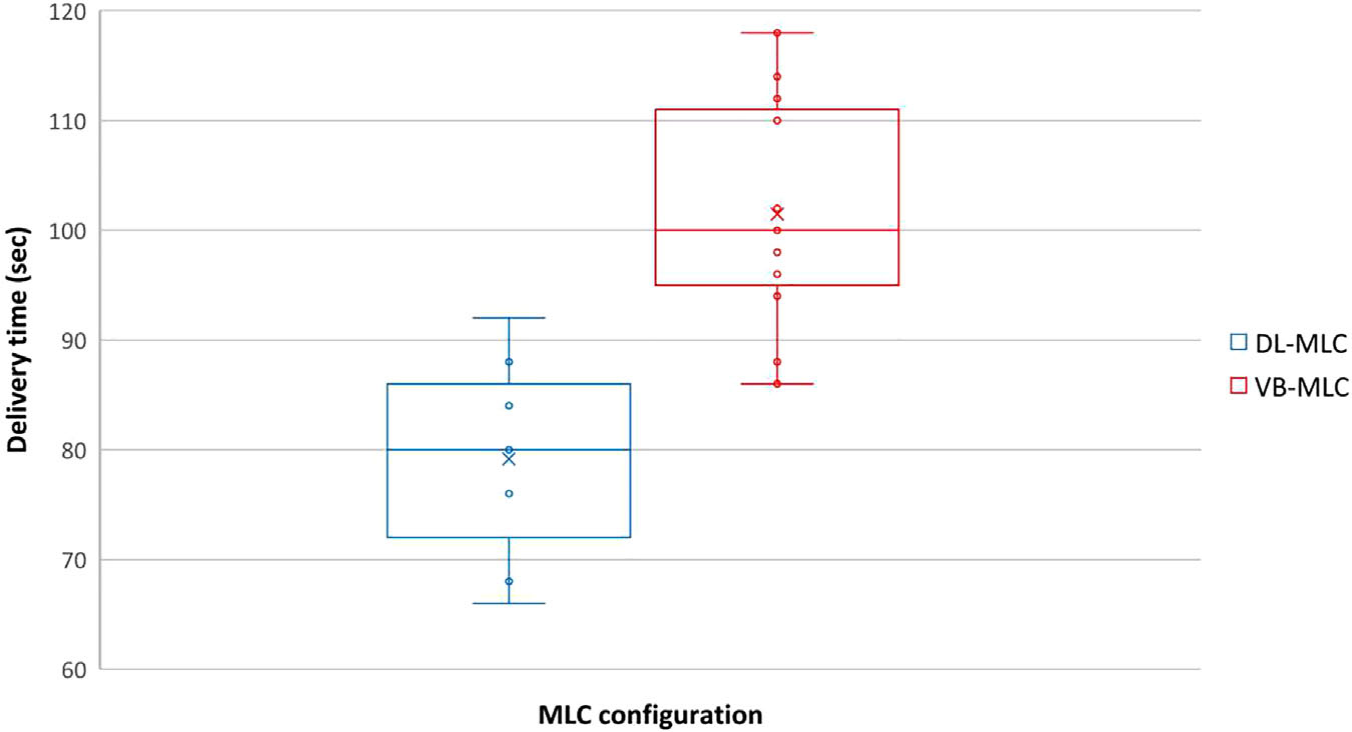
Box plot for the delivery time of DL‐MLC and VB‐MLC

The delivery times are compared in Figure 4. Specifically, the delivery times for the DL‐MLC and VB‐MLC plans were 79 ± 10 and 101 ± 11 s, respectively. The average delivery time of DL‐MLC was 27.8% lower than that of VB‐MLC.

## 4 DISCUSSION

In this study, to treat early‐stage left‐sided breast cancer, we compared the plan quality evaluations of DL‐MLC and VB‐MLC plans in terms of target coverage and OAR sparing. We found that the DL‐MLC plans led to some improvement in OAR sparing. Furthermore, it is presumed that the differences in the MLC configuration between the two MLC systems induced dosimetric differences in the OARs.

The leaf size of the novel staggered DL‐MLC in Halcyon^TM^ (1 cm) was larger than that of the Millennium 120‐leaf MLC in VitalBeam^®^ (0.5 cm). Furthermore, the DL‐MLC entails less 6‐MV‐FFF transmission compared with the Millennium 120‐leaf MLC (0.01% and 1.36%, respectively). The DLG is 0.01 mm in the DL‐MLC, in contrast to the 0.5‐mm DLG in the Millennium 120‐leaf MLC.^16^ Thus, recent studies focused on the impact of MLC parameters on plan quality. Li et al. compared DL‐MLC plans to Millennium 120‐leaf MLC plans for nine patients with head and neck cancer. Compared with the Millennium 120‐leaf MLC plans for IMRT, the DL‐MLC plans exhibited improved OAR sparing regarding mean doses to the esophagus and larynx (*p* < 0.01, *p* = 0.03, respectively). Furthermore, the DL‐MLC plans demonstrated marginal OAR sparing for the left and right cochlea (*p* = 0.06, *p* = 0.06, respectively).^16^ In another study, Li et al. compared the Halcyon^TM^ DL‐MLC to the Millennium 120‐leaf MLC with Trilogy for 30 cervical carcinoma patients. For the PTVs, the two plans were comparable in terms of *D*_2%_, *D*_98%_, HI, CI, and the gradient index. However, *V*_40Gy_ to the bladder was significantly different between the DL‐MLC plans and Millennium 120‐leaf MLC plans (24.37% ± 6.87% and 28.41% ± 7.62%, respectively; *p* = 0.001).^17^ In our study, the two plans (DL‐MLC and VB‐MLC) were comparable in terms of QC, CI, and HI for the PTVs. The *V*_30Gy_ value of the heart and *V*_20Gy_ of the whole lung in the DL‐MLC plan were significantly lower than those in the VB‐MLC plan (*V*_30Gy_ = 0.256% ± 0.498% and 0.746% ± 0.697%, respectively; *p* < 0.001; *V*_20Gy_ = 5.065% ± 0.888% and 5.823% ± 1.060%, respectively; *p* = 0.010).

Some studies have compared dosimetric differences between plans of breast cancer with those of Halcyon^TM^ and other conventional linac. Sun et al. compared the dosimetric difference between different arc plans with Halcyon^TM^ and Trilogy,^20^ with the prescription dose of 50 Gy per 25 fractions to the PTV. For the heart, Halcyon^TM^ 4arc‐plans reduced the *V*_30Gy_ value; however, *D*_mean_ increased compared with Trilogy 8arc‐plans (*V*_30Gy_ = 0.8% ± 0.5%, 1.8 ± 1.7%; *D*_mean_ = 5.6 ± 0.6, 3.9 ± 1.1 Gy). *D*_mean_ of the lungs significantly increased for Halcyon^TM^ 4arc‐plans compared with Trilogy 8arc‐plans (*D*_mean_ = 9.1% ± 0.8%, 7.5% ± 1.0%). Morris et al. compared the dosimetric difference between Halcyon^TM^ plans and TruBeam plans,^21^ for a prescription dose of either 4256 cGy per 16 fractions or 5040 cGy per 28 fractions to the PTV. *D*_mean_ of the heart increased for Halcyon^TM^ field‐in‐field plans compared with TrueBeam field‐in‐field plans (*D*_mean_ = 149.58 ± 129.01, 97.96 ± 78.27 cGy). However, *D*_mean_ of the ipsilateral lung decreased for Halcyon^TM^ field‐in‐field plans compared with TrueBeam field‐in‐field plans (*D*_mean_ = 569.44 ± 221.73, 676.34 ± 175.84 cGy). In our study, *V*_30Gy_ and *D*_mean_ of the heart significantly reduced for the Halcyon^TM^ 2arc‐plans compared with 2arc VitalBeam^®^ plans (*V*_30Gy_ = 0.256% ± 0.498%, 0.746% ± 0.697%, *p* < 0.001; *D*_mean_ = 549.024 ± 61.894, 642.656 ± 65.445 cGy, *p* < 0.001). Moreover, there were significant differences in *D*_mean_ of the ipsilateral lung between the DL‐MLC and VB‐MLC plans (949.088 ± 74.144 and 1004.416 ± 74.652 cGy). Statistically significant differences were found in the *D*_mean_ and *V*_20Gy_ values of the whole lungs between the DL‐MLC plans and VB‐MLC plans (*D*_mean_ = 621.376 ± 42.687, 659.680 ± 39.908 cGy, *p* < 0.001; *V*_20Gy_ = 5.065% ± 0.888%, 5.823% ± 1.060%, *p* = 0.010).

Radiotherapy for early‐stage breast cancer reduces the rate of recurrence and death. However, this regimen can lead to an increased risk of ischemic heart disease via incidental irradiation of the heart. The rates of major coronary events increased by 7.4% per Gy increase in the mean dose to the heart.^13^ Additionally, the risk of major coronary events increased by 6.4% per Gy increase in the mean dose to the heart.^14^ In our study, the mean dose to the heart in the DL‐MLC plan was significantly reduced by 14.6%, compared with that in the VB‐MLC plan (549.024 ± 61.894 and 642.656 ± 65.445 cGy, respectively; *p* < 0.001). Therefore, for left‐sided breast cancer, the risk of heart toxicity is decreased in the case of treatment based on the DL‐MLC plan.

## 5 CONCLUSION

In this study, we compared the plan quality between the DL‐MLC and VB‐MLC plans. While there were differences in mechanical characteristics (i.e., lower transmission, lower DLG, faster gantry speed, and faster collimator speed), DL‐MLC plans exhibited improved OAR sparing. In particular, when treating left‐sided breast cancer via DL‐MLC plans, the risk of heart toxicity is expected to be reduced.

## CONFLICT OF INTEREST

No conflicts of interest exist. This study had no sponsor involvement.

## AUTHOR CONTRIBUTIONS

Conceived and designed the analysis: EBJ, SL. Collected the data: EBJ. Performed the analysis: CGP, MK. Validation: KHK, JBS, YJP, NKL, CYK. Wrote the draft paper: EJH. Supervision: SL. Writing‐review & editing: KHK, JBS, YJP, NKL, CYK. Final approval of the version to be published: all authors.
